# Assessing the Dependence and Perceptions of the Harm and Addictiveness of Electronic Cigarettes among Saudi University Students: A Cross-Sectional Study

**DOI:** 10.3390/healthcare12131289

**Published:** 2024-06-27

**Authors:** Abdulwahab Aqeeli, Abdullah A. Alsabaani, Hassan Alshaiban, Ahmad Y. Alqassim, Anwar S. Alahmar, Abdullah Sabai, Saud Alwadani

**Affiliations:** 1 Department of Family and Community Medicine, Faculty of Medicine, Jazan University, Jazan 45142, Saudi Arabia; aalqassim@jazanu.edu.sa; 2 Saudi Board for Preventive Medicine Residency Program, King Khalid University, Abha 61913, Saudi Arabia; 3 Department of Family and Community Medicine, College of Medicine, King Khalid University, Abha 61421, Saudi Arabia; aalsabaani@kku.edu.sa; 4 Preventive Medicine Program, Aseer Health Cluster, Abha 62523, Saudi Arabia; hassana@moh.gov.sa; 5 ERADH and Mental Health Hospital, Jazan Health Cluster, Jazan 82943, Saudi Arabia; aalahmar@moh.gov.sa; 6 Population Health Unit, Jazan Health Affairs, Jazan 82611, Saudi Arabia; asuba@moh.gov.sa; 7 Faculty of Medicine, Jazan University, Jazan 45142, Saudi Arabia; 202005240@stu.jazanu.edu.sa

**Keywords:** electronic cigarettes, dependence, university students, addiction, smoking cessation, public health

## Abstract

(1) Background: The rising prevalence of e-cigarette use among university students necessitates a comprehensive understanding of dependence levels and associated factors. This study investigates e-cigarette dependence among Jazan University students in Saudi Arabia. (2) Methods: With the use of a cross-sectional design, data were collected from 1187 students through an online survey from January to April 2024. Dependence levels were assessed using the Penn State Electronic Cigarette Dependence Index (PS-ECDI), and the Arabic version of the questionnaire was validated through pilot testing. Multivariable logistic regression analysis was employed to identify factors associated with e-cigarette dependence. (3) Results: The results indicated that among current e-cigarette users, 37.4% had low dependence, 34.4% had medium dependence, and 13.8% had high dependence. A significant proportion of current and ever users regarded e-cigarettes to be less harmful (50.6% and 44.4%, respectively) and less addictive (37.9% and 32.3%, respectively) than cigarettes. Significant factors influencing dependence included gender, with males showing higher odds of medium (AOR = 12.8, 95% CI: 3.4–47.6) and low dependence (AOR = 9.7, 95% CI: 2.5–37.3) compared to females. Longer duration of e-cigarette use (>2 years) was strongly associated with high dependence (AOR = 50, 95% CI: 7.7–324). Daily use and multiple quit attempts were also significant predictors of higher dependence levels. (4) Conclusions: These findings highlight the substantial e-cigarette dependence among university students and underscore the need for targeted interventions to address this growing public health concern.

## 1. Introduction

Electronic cigarettes (e-cigarettes) have surged in popularity among youth and young adults in recent years. E-cigarettes are battery-powered devices that heat a liquid, usually containing nicotine and flavorings, to generate an aerosol for inhalation [1]. Nicotine exposure can lead to dependence, and emerging research indicates concerning levels of e-cigarette dependence among adolescents and university students [2,3]. However, studies on e-cigarette dependence among university students in Saudi Arabia are lacking.

E-cigarette use has risen exponentially among adolescents and young adults globally. In the U.S., according to a study using data from the Behavioral Risk Factor Surveillance System (BRFSS), the age-standardized prevalence of current e-cigarette use among adults was 6.9%. Notably, among individuals aged 18 to 24 years, the prevalence of current e-cigarette use was 18.6%, with 9.0% reporting daily use [4]. Moreover, past 30-day e-cigarette use increased from 1.5% in 2011 to 20.8% in 2018 among high school students [5]. Likewise, ever e-cigarette use rose from 4.9% in 2013 to 10.8% in 2016 among university students in the U.K. [6]. A study among university students in Jordan reported a lifetime prevalence of e-cigarette use at 14% [7]. In Saudi Arabia, some studies have estimated prevalence use of e-cigarettes up to 40% among college students [8,9,10,11].

In addition to traditional cigarette smoking, e-cigarette use has become increasingly prevalent among students in recent years. Epidemiological trends reveal a paradoxical shift in adolescent nicotine consumption patterns. While traditional tobacco use has experienced a decline, there has been a concomitant and significant surge in e-cigarette adoption among this demographic [12]. Research on medical students in Western Balkan countries did not specifically examine e-cigarette use but noted the importance of considering new tobacco products in future studies. The rising popularity of e-cigarettes among young adults, including health profession students, highlights the need to include these devices in assessments of tobacco use behaviors and attitudes [13]. However, data on e-cigarette use specifically among dental students remains limited, representing a gap in the current literature.

University students are a high-risk population for e-cigarette initiation and dependence due to several factors. The age of matriculation into higher education coincides with a high-risk period of experimentation and substance use behaviors [14,15,16]. Perceptions of e-cigarette harmfulness are lower compared to cigarettes, promoting experimentation among student groups [17]. Additionally, adjusting to stressors among adolescents and young adults may also prompt e-cigarette adoption as a coping mechanism [18,19]. Influential factors include perceived safety, social influences, marketing, and flavor variety. While some use e-cigarettes as a cessation tool, dual use of both e-cigarettes and regular cigarettes is common. A study in Poland reported an increase in cigarette smoking among e-cigarette users [20].

Using electronic cigarettes carries significant health issues including respiratory and cardiovascular disorders. Studies have shown that e-cigarette use can cause lung inflammation, decreased immunological responses, and an increased susceptibility to infection. Furthermore, the chemicals and nicotine in e-cigarettes have been related to elevated heart rate and blood pressure, which raise the risk of heart disease [21]. Moreover, smoking has been linked to poorer academic performance among students, as nicotine dependence and smoking-related health issues can lead to decreased concentration, cognitive impairments, and increased absenteeism [22].

Dependence on e-cigarettes has been associated with difficulty quitting or transitioning completely away from nicotine and tobacco products. Among U.S. adolescent e-cigarette users, greater dependence measured by the Penn State Electronic Cigarette Dependence Index was significantly associated with failure of past year quit attempts [23]. Furthermore, e-cigarette dependence appears to impede smoking cessation in adult smokers. A clinical trial found e-cigarette users with high dependence had significantly lower smoking abstinence compared to those with minimal dependence [24].

A limited number of studies have assessed the level of e-cigarette dependence among youth and young adults using validated instruments. A study reported a mean dependence score of 7.4 (range 0–13) among high school e-cigarette users, indicating a moderate level of dependence [24]. Among U.S. college students, a study found mean dependence scores of 5.2 and 6.5 for ever and past 30-day e-cigarette users [25]. In a young adult sample in Mexico, 58.6% screened positive for dependence based on feeling cravings, withdrawal, and difficulty quitting e-cigarettes [26]. Outside North America and Western countries, research on e-cigarette dependence is sparse. One study in Kuwait administered an Arabic translation of the Penn State index and found 84.8% of ever e-cigarette users exhibited dependence [2]. Our study stands out by focusing on e-cigarette dependence among university students in Saudi Arabia, a population and context that have been largely overlooked in previous research. The main objectives of this study are to measure the level of electronic cigarette dependence, to estimate the prevalence of current and ever electronic cigarette use, to assess the perception of the harm and addictiveness of e-cigarettes compared to cigarettes, and to investigate factors associated with the levels of electronic cigarette dependence among university students in Jazan, Saudi Arabia.

## 2. Materials and Methods

### 2.1. Study Design, Setting, and Population

An analytical observational cross-sectional study was conducted from January to April 2024 utilizing a self-administered survey. The study population comprised a sample of undergraduate students at Jazan University in Jazan, Saudi Arabia, registered during the 2023–2024 academic year. The study was conducted at Jazan University, which is situated in the city of Jazan and is the main center of higher education in the province. Jazan University has an enrollment exceeding 50,000 students distributed across 26 colleges and campuses covering diverse academic disciplines. Data collection was conducted at the selected colleges after obtaining necessary permissions. The diversity of Jazan University’s student population and programs provides an ideal setting to survey students from different fields of study. Ethical approval (REC-45/04/829) of the study was obtained from the Standing Committee for Scientific Research-Jazan University.

### 2.2. Sample Size and Sampling Technique

The target sample size is 1258 students. This was determined based on the estimated e-cigarette prevalence in Saudi Arabia of 21% [11], a margin of error of 3%, a design effect set at 1.5, a confidence level of 95%, and a non-response rate of 20%.

A stratified random sampling technique was utilized. First, Jazan University colleges were categorized into three strata based on broad fields of study—health, science, and other. Two colleges were randomly selected from each stratum. Then, probability proportional to size sampling was applied to determine the number of students sampled from each selected college. Finally, within the chosen colleges, classes were randomly selected, and all students present in those classes on the day of data collection were invited to participate.

### 2.3. Data Collection Tool

Data were collected via a structured self-administered electronic questionnaire. The questionnaire collected information about the sociodemographic characteristics of the sample, e-cigarette and cigarette use, the perception of e-cigarette harm and addictiveness, and the level of e-cigarette use dependence. Assessment of the sociodemographic included age, gender, year of study, type of residence, academic performance, family income, and marital status.

The dependence level was assessed using the Penn State Electronic Cigarette Dependence Index (PS-ECDI) [3], which was originally developed in English. This index was translated into Arabic by two study investigators who are native Arabic speakers and fluent in English. Other investigators checked the Arabic translation to ensure its coherence with the original version. The Arabic version of the questionnaire was then tested in a pilot study. The questionnaire’s clarity and coherence were also assessed. This validated 10-item measure was used to generate each participant’s total dependence score, which ranged from 0 to 20. Based on the scoring system devised by Foulds and colleagues [3], the classifications are as follows: not dependent (scores ranging from 0 to 3), low dependence (scores between 4 and 8), medium dependence (scores from 9 to 12), and high dependence (scores of 13 or higher).

The perception of the harm of e-cigarettes relative to cigarettes was assessed by asking a single question: ‘Compared to cigarettes, what is the level of harm caused by electronic cigarettes?’ The perception of the addictiveness of e-cigarettes compared to cigarettes was assessed with the question: ‘Compared to cigarettes, what is the potential for addiction to electronic cigarettes?’ The students were also asked about ever e-cigarette use as having used an e-cigarette at least once and ‘current use’ as having used e-cigarettes for at least 1 day in the last 30 days.

### 2.4. Data Processing and Analysis

Data were collected electronically through a link to Google Forms. Data were exported from the Google Forms platform to an Excel sheet for review and coding. After coding, data were transferred to Stata software version 16 (StataCorp, College Station, TX, USA) for final analysis.

The statistical significance level for all association analyses was set at α = 0.05. Descriptive analyses were used to determine the frequencies and percent of categorical variables, including the categories for e-cigarette dependency. The score of the dependence on e-cigarettes, a quantitative variable that does not follow a normal distribution, was summarized using the median and interquartile range (IQR). A chi-squared (χ^2^) test was used to see if the prevalence of ever and current e-cigarette usage varied across different sociodemographic categories, except for age, which was tested using the Mann–Whitney test. With the use of ‘no dependence’ as the reference category, a multinomial logistic regression model was used to examine the relationship between different factors (independent variables) and the level of e-cigarette dependence (ordinal outcome variable). The model produced adjusted odds ratios (AORs) and their 95% confidence intervals (CIs).

## 3. Results

A total of 1187 students participated in our study, of whom 703 (59.2%) were males and 484 (40.8%) were females (Table 1). The participants’ median age was 21 years, with an interquartile range (IQR) of 20 to 22 years. Most participants were living in rural areas (54.8%) and enrolled in their fourth year of study (29.4%). Of the study sample, 40.5% reported having excellent academic performance and 40.1% reported very good performance. There were almost equal percentages of enrollment among the three college fields, with 35.6% for the health-related field, 31% for the science field, and 33.4% for other fields of study.

Of the total study participants, 4.9% were former cigarette smokers and 7.2% were recorded as current cigarette smokers (Table 1). Overall, the prevalence of ever e-cigarette use was 22.4%, with 14.7% being current users of e-cigarettes (Table 1).

The prevalence of current e-cigarette use did not differ between males and females (16.2% vs. 12.4%, *p* = 0.067) (Table 1). However, there was a statistically significant difference in the prevalence of current e-cigarette use according to residence, year of study, academic performance, and body mass index. Current cigarette smokers were significantly more likely to be current e-cigarette users compared to never smokers (55.3% vs. 11.2%, *p* < 0.001) (Table 1).

Table 2 presents the distribution of beliefs and perceptions about e-cigarettes among the study participants, categorized by their e-cigarette use status (never, ever, and current users). When examining the belief that e-cigarette users are smokers, 88.2% of the total sample held this belief. Specifically, 91.5% of never e-cigarette users, 76.7% of ever e-cigarette users, and 77.6% of current e-cigarette users believed that e-cigarette users are smokers (Table 2). Participants were also asked if they believed e-cigarettes could be used to quit smoking. Overall, 16% of the total sample believed e-cigarettes could aid in quitting smoking. This belief was more prevalent among ever users (38.7%) and current users (42%) compared to never users (9.5%) (Table 2).

Perceptions of the harm caused by e-cigarettes relative to cigarettes varied among participants. Of the total sample, 20.8% believed e-cigarettes were less harmful, 32.6% believed they were equally harmful, 23.3% believed they were more harmful, and 23.3% did not know. Notably, a higher proportion of current users (50.6%) and ever users (44.4%) considered e-cigarettes to be less harmful compared to never users (14%) (Table 2).

Regarding the addiction potential of e-cigarettes compared to cigarettes, 20.1% of the total sample believed e-cigarettes were less addictive, 42.5% believed they were equally addictive, 16% believed they were more addictive, and 21.4% did not know. Among current e-cigarette users, 37.9% perceived e-cigarettes as less addictive while 32.3% of ever users and 16.6% of never users shared this belief (Table 2).

Table 3 provides an overview of the reasons for using e-cigarettes, self-perceived dependence, and Penn State Electronic Cigarette Dependence Index (PS-ECDI) levels among ever and current e-cigarette users. Overall, the median PS-ECDI score for ever users of e-cigarettes was 6.0 (IQR: 5.0–11.0) while for current users it was higher at 8.0 (IQR: 5.0–11.0). Among current users, 14.4% were not dependent, 37.4% had low dependence, 34.4% had medium dependence, and 13.8% had high dependence. When asked about their self-perceived dependence on e-cigarettes, 35.6% of current users felt they were not dependent, 37.4% felt they were somewhat dependent, and 27% felt they were highly dependent.

The reasons for using e-cigarettes among current users varied. The most common reasons included a friend or family member using them (59.8%), the availability of different flavors (54.6%), and curiosity (32.8%). Other reasons were the ability to use them unnoticed (31%), the perception that they are less harmful than cigarettes (28.2%), ease of access (28.2%), and using them to quit smoking (19.5%).

Adjusted associations between different factors and e-cigarette dependence levels are shown in Table 4. Gender (male vs. female) was associated with low dependence (AOR = 9.7; 95% CI: 2.5–37.3) and medium dependence (AOR = 12.8; 95% CI: 3.4–47.6) levels. Moreover, using e-cigarettes for >2 years compared to ≤2 years was associated with high dependence (AOR = 50; 95% CI: 7.7–324). Compared to those who never tried to stop using e-cigarettes, participants who tried to stop e-cigarette use one to three times in the past 12 months were more likely to be highly dependent (AOR = 50; 95% CI: 6–411). When other variables were adjusted for, cigarette smoking and the use of e-cigarettes in the past 30 days were not associated with level of dependence of e-cigarette use.

## 4. Discussion

In this study, ever (22.4%) and current (14.7%) e-cigarette use was high among university students in Jazan, Saudi Arabia. These results support Saudi Arabian studies that found over 21% of adolescents ever used e-cigarettes [11]. The results also suggest that e-cigarette use may be more common than conventional cigarette smoking among university students in our context. This aligns with global trends indicating a rise in e-cigarette use among youth and young adults, often surpassing traditional cigarette smoking rates [27,28]. However, it is important to note that a significant proportion of e-cigarette users in our study (55.3%) were also current cigarette smokers, highlighting the issue of dual use. This finding is consistent with that of previous research demonstrating that e-cigarette use does not necessarily lead to a reduction in conventional cigarette smoking and may even contribute to an increase in overall tobacco product use [20,29]. The higher prevalence of e-cigarette use compared to cigarette smoking among university students in Jazan underscores the need for targeted interventions and policies to address this emerging public health concern and prevent the potential gateway effect to other tobacco products. University students are at high risk for e-cigarette addiction, so our study’s findings are concerning [6,18]. University prevention efforts are needed because the age of matriculation into higher education coincides with a critical period of experimentation and substance use [6].

Notably, our study found subgroup differences in e-cigarette use. Current e-cigarette use did not differ between males and females, but there were differences based on residence, academic year, academic performance, and BMI. E-cigarette use is higher among urban residents, upper academic years, and those with lower academic performance, suggesting environmental and academic factors may influence usage. E-cigarette use also rises with higher BMI, supporting previous research linking it to weight control [19]. Compared to the U.S. [12] and U.K. [6], our prevalence rates suggest cultural or regional factors influencing Saudi Arabian e-cigarette use. Sociocultural norms, accessibility, and regulatory environments should be studied to inform prevention strategies.

Our study revealed university students’ concerns about e-cigarette harm and addiction compared to conventional cigarettes. E-cigarettes were perceived as less harmful (50.6% and 44.4%, respectively) and less addictive (37.9% and 32.3%, respectively) than conventional cigarettes, according to a substantial proportion of current and ever users. These findings support those of previous research showing that e-cigarettes are misunderstood as safer than conventional cigarettes [28,29]. Additionally, 16% of the sample believed that e-cigarettes could help to quit smoking, with higher rates of belief among current users. This contradicts evidence that e-cigarettes may hinder smoking cessation, especially in highly dependent smokers [24]. E-cigarette initiation and use among university students are affected by widespread beliefs and perceptions about their reduced harm and addictiveness [30]. These misconceptions may contribute to the normalization and acceptability of e-cigarette use in this population, encouraging experimentation and long-term use.

Despite evidence suggesting otherwise, e-cigarettes may be used by smokers trying to quit [24]. These findings highlight the need for comprehensive educational campaigns targeting university students to address misconceptions and raise awareness of e-cigarette risks. Such campaigns should dispel e-cigarette safety and addictiveness myths, emphasize their potential harm, and provide accurate information on evidence-based smoking and e-cigarette cessation strategies.

Our study revealed concerning levels of e-cigarette dependence among university students in Jazan, Saudi Arabia. The Penn State Electronic Cigarette Dependence Index (PS-ECDI) showed that 85.6% of e-cigarette users were dependent. This finding suggests that e-cigarettes pose a significant public health risk among Saudi students, necessitating the development of preventive initiatives. The difference between self-perceived and objective dependence levels was notable. Although 35.6% of current users considered themselves non-dependent, the PS-ECDI classified only 14.4% as such, suggesting that this population may understate their dependence. Since self-perception may differ from objective measures, validated instruments are needed to accurately assess e-cigarette dependence. 

Our multivariable analysis found significant associations between e-cigarette dependence and gender, duration, and quit attempts. Male students had higher levels of low (AOR = 9.7; 95% CI: 2.5–37.3) and medium (AOR = 12.8; 95% CI: 3.4–47.6) dependence than females. E-cigarette use over two years was associated with high dependence (AOR = 50; 95% CI: 7.7–324). Moreover, we found that students who tried to quit e-cigarettes one to three times in the past year were more likely to be highly dependent (AOR = 50; 95% CI: 6–411). These findings emphasize the need for targeted interventions to prevent and address e-cigarette dependence in university students, particularly for at-risk subgroups like male students and long-term users. The association between quit attempts and higher dependence levels suggests that dependent e-cigarette users need tailored cessation support and strategies.

Our study found that university students in Jazan, Saudi Arabia, use e-cigarettes for many reasons. Current e-cigarette users most often reported a friend or family member using them (59.8%), flavor availability (54.6%), and curiosity (32.8%). These findings support those of previous research that shows peer influences, appealing flavors, and novelty encourage youth and young adult e-cigarette experimentation and use [31,32]. Our study’s diverse e-cigarette motivations have important implications for university student prevention and cessation strategies. Peer and social factors emphasize the need for comprehensive interventions that address the social determinants of e-cigarette use in this age group. Educational campaigns and peer-led initiatives that challenge normative beliefs and promote healthy behaviors may counteract social influences. Flavored e-cigarettes, which are popular with youth and young adults, should be regulated [33,34]. The reported perceptions of reduced harm and discreetness emphasize the need for targeted educational efforts to dispel e-cigarette myths about risks and harms.

Our study demonstrates the urgent need for comprehensive policies and regulations to address the Saudi e-cigarette epidemic among youth and young adults. Our study found high rates of e-cigarette use and worrying levels of dependence among university students, emphasizing the need to prevent their use. Targeted educational campaigns and campus-based cessation programs tailored to e-cigarette users can raise awareness of e-cigarette risks, dispel myths about their safety and addictiveness, and help users quit [6]. Restricting e-cigarette marketing and flavored products, which youth and young adults find appealing, may also reduce e-cigarette use [35]. Our findings emphasize the need to address e-cigarette dependence as part of smoking cessation efforts, as high dependence levels have been linked to lower quit rates and may hinder success [24]. To help dependent e-cigarette users quit, tailored interventions may be needed to address withdrawal symptoms and cravings.

This study has some limitations that should be considered when interpreting the findings. The cross-sectional design of the study does not establish causal relationships, and longitudinal studies are needed to understand the temporal relationships. The data were collected through self-reported measures, which may be subject to social desirability bias and recall inaccuracies. Future research could incorporate biochemical verification and objective measures of dependence. The study was conducted at a single university, which may limit its generalizability to other settings or regions. Cultural and contextual factors may influence e-cigarette use patterns and dependence levels. This study did not explore the potential dual or poly-use of other tobacco or nicotine products. The validated measure of e-cigarette dependence (PS-ECDI) was initially validated among adult users, but further research is needed to evaluate its applicability among university student populations. Despite these limitations, this study contributes valuable insights into the prevalence, perceptions, and dependence levels associated with e-cigarette use among university students in Saudi Arabia, highlighting the need for targeted interventions and policies to address this public health concern.

## 5. Conclusions

This study reveals a significant prevalence of e-cigarette dependence among Jazan University students, with a substantial proportion of current e-cigarette users exhibiting medium and high levels of dependence. Key factors influencing dependence include gender, duration of e-cigarette use, daily usage, and previous quit attempts. Males and those with longer use durations are particularly at risk for higher dependence levels. These findings emphasize the urgent need for targeted public health interventions and educational programs to address e-cigarette use and dependence in this population, highlighting the importance of tailored strategies to mitigate the risks associated with prolonged e-cigarette use among university students. Additionally, the perception of e-cigarettes as less harmful than traditional cigarettes may contribute to their widespread use and the resulting dependence, necessitating efforts to correct these misconceptions.

## Figures and Tables

**Table 1:** Characteristics of the study sample and prevalence of ever and current use of electronic cigarettes (e-cigarettes) among Jazan University students in Saudi Arabia.

Variable	Total n (%)	Ever Use of e-Cigarettes		Current Use of e-Cigarettes	
		n (%)	*p*-Value *	n (%)	*p*-Value *
Overall sample	1187 (100)	266 (22.4)	-	174 (14.7)	-
Median age (IQR)	21 (20–22)	22 (20–23)	**<0.001**	22 (20–23)	**<0.001**
**Gender**					
Male	703 (59.2)	190 (27)	**<0.001**	114 (16.2)	0.067
Female	484 (40.8)	76 (15.7)		60 (12.4)	
**Residence**					
Urban	537 (45.2)	153 (28.5)	**<0.001**	119 (22.2)	**<0.001**
Rural	650 (54.8)	113 (17.4)		55 (8.5)	
**Year of study**					
First	279 (23.5)	39 (14)	**<0.001**	21 (7.5)	**<0.001**
Second	181 (15.3)	43 (23.8)		29 (16)	
Third	169 (14.2)	70 (41.4)		43 (25.4)	
Fourth	349 (29.4)	61 (17.5)		43 (12.3)	
Fifth	139 (11.7)	46 (33.1)		38 (27.3)	
Sixth	70 (5.9)	7 (10)		0 (0)	
Academic performance **					
Excellent	480 (40.5)	96 (20)	**<0.001**	76 (15.8)	**0.001**
Very good	476 (40.1)	134 (28.2)		83 (17.4)	
Good	189 (15.9)	27 (14.3)		10 (5.3)	
Pass	42 (3.5)	9 (21.4)		5 (11.9)	
**College field**					
Health	422 (35.6)	104 (24.6)	0.111	57 (13.5)	0.624
Non sciences	396 (33.4)	93 (23.5)		63 (15.9)	
Sciences	369 (31)	69 (18.7)		54 (14.6)	
**Body mass index**					
Underweight	192 (16.2)	45 (23.4)	**<0.001**	18 (9.4)	**<0.001**
Normal weight	656 (55.3)	134 (20.4)		82 (12.5)	
Overweight	249 (21)	45 (18.1)		42 (16.9)	
Obese	90 (7.5)	42 (46.7)		32 (35.6)	
**Cigarette smoking status**					
Current smoker	85 (7.2)	67 (78.8)	**<0.001**	47 (55.3)	**<0.001**
Ex-smoker	58 (4.9)	25 (43.1)		10 (17.2)	
Never	1044 (87.9)	174 (16.7)		117 (11.2)	

Note: Values in parentheses are the row percentages. * Based on chi-squared (χ^2^) test except for age, which was tested using the Mann–Whitney test. ** Categories based on the following grade point average (GPA) scale: Excellent = 4.5–5.0, Very good = 3.75–4.49, Good = 3.0–3.74, Pass = 2.0–2.99. Bold *p*-values indicate statistical significance at the *p* < 0.05 level.

**Table 2:** Perception of considering an e-cigarette user as a smoker, e-cigarettes as a smoking cessation aid, and harmfulness and dependence potential of e-cigarettes compared to cigarettes.

Variable	Total Sample N = 1187	Never Use of e-Cigarette N = 921	Ever Use of e-Cigarette N = 266	Current Use of e-Cigarettes N = 174
	n (%)	n (%)	n (%)	n (%)
**Believe that an e-cigarette user is a smoker**				
Yes	1047 (88.2)	843 (91.5)	204 (76.7)	135 (77.6)
No	140 (11.8)	78 (8.5)	62 (23.3)	39 (22.4)
**Believe that e-cigarettes can be used to quit smoking**				
Yes	190 (16)	87 (9.5)	103 (38.7)	73 (42)
No	997 (84)	834 (90.5)	163 (61.3)	101 (58)
**E-cigarette harm compared to cigarettes**				
Less harmful	247 (20.8)	129 (14)	118 (44.4)	88 (50.6)
Equally harmful	387 (32.6)	330 (35.8)	57 (21.4)	40 (23)
More harmful	276 (23.3)	225 (24.5)	51 (19.2)	31 (17.8)
Do not know	277 (23.3)	237 (25.7)	40 (15)	15 (8.6)
**E-cigarette dependence compared to cigarettes**				
Less addictive	239 (20.1)	153 (16.6)	86 (32.3)	66 (37.9)
Equally addictive	504 (42.5)	390 (42.4)	114 (42.9)	76 (43.7)
More addictive	190 (16)	156 (16.9)	34 (12.8)	22 (12.6)
Do not know	254 (21.4)	222 (24.1)	32 (12)	10 (5.8)

Note: Values in parentheses are the column percentages.

**Table 3:** Reasons for using e-cigarettes, self-perceived dependence, and PS-ECDI levels among ever and current users of e-cigarettes.

Variable	Ever Use of e-Cigarettes N = 266 n (%)	Current Use of e-Cigarettes N = 174 n (%)
PS-ECDI score median (IQR)	6.0 (5.0–11.0)	8.0 (5.0–11.0)
**PS-ECDI level**		
Not dependent	35 (13.2)	25 (14.4)
Low	134 (50.4)	65 (37.4)
Medium	73 (27.4)	60 (34.4)
High	24 (9)	24 (13.8)
**Self-perceived e-cigarette dependence**		
Not dependent	154 (57.9)	62 (35.6)
Somewhat dependent	65 (24.4)	65 (37.4)
Highly dependent	47 (17.7)	47 (27)
**Reasons for using e-cigarettes**		
A friend or a family member is using	152 (57.1)	104 (59.8)
Have different flavors	139 (52.3)	95 (54.6)
Can be used unnoticed	89 (33.5)	54 (31)
Less harmful than cigarettes	88 (33.1)	49 (28.2)
Out of curiosity	86 (32.3)	57 (32.8)
Easy to obtain	59 (22.2)	49 (28.2)
To quit smoking	50 (18.8)	34 (19.5)

Note: Values in parentheses are column percentages. Abbreviation: PS-ECDI = Penn State Electronic Cigarette Dependence Index; IQR = interquartile range.

**Table 4:** Factors associated with e-cigarette dependence levels among Jazan university student users in Saudi Arabia: Multivariable analysis.

Factor	Level of e-Cigarette Dependence AOR (95% CI)			
	Not Dependent	Low	Medium	High
**Gender**				
Male vs. female	Ref.	9.7 (2.5–37.3) *	12.8 (3.4–47.6) *	2.3 (0.5–11.8)
**Smoking cigarettes**				
Smoker vs. non-smoker	Ref.	0.5 (0.1–1.8)	0.6 (0.2–2.1)	0.3 (0.1–1.7)
**Years of using e-cigarettes**				
>2 vs. ≤2	Ref.	0.1 (0.01–0.4)	2 (0.5–7.7)	50 (7.7–324) *
**Use of e-cigarettes in previous 30 days**				
Daily vs. non-daily	Ref.	2.4 (0.8–7.5)	1.2 (0.4–3.9)	0.3 (0.1–1.4)
**Quit attempts in the past 12 months**				
1–3 vs. never	Ref.	0.4 (0.1–1.6)	2.7 (0.6–12.3)	50 (6.2–411) *
≥3 vs. never	Ref.	0.3 (0.1–1.4)	1.1 (0.2–5.2)	4.2 (0.6-29.2)

Abbreviations: AOR = Adjusted Odds Ratio; CI = Confidence Interval. * Statistically significant.

## Data Availability

The data that support the findings of this study are available from the corresponding author upon reasonable request.
