# Correction: Aqeeli et al. Assessing the Dependence and Perceptions of the Harm and Addictiveness of Electronic Cigarettes among Saudi University Students: A Cross-Sectional Study. *Healthcare* 2024, *12*, 1289

**DOI:** 10.3390/healthcare13050450

**Published:** 2025-02-20

**Authors:** Abdulwahab Aqeeli, Abdullah A. Alsabaani, Hassan Alshaiban, Ahmad Y. Alqassim, Anwar S. Alahmar, Abdullah Sabai, Saud Alwadani

**Affiliations:** 1 Department of Family and Community Medicine, Faculty of Medicine, Jazan University, Jazan 45142, Saudi Arabia; aalqassim@jazanu.edu.sa; 2 Saudi Board for Preventive Medicine Residency Program, King Khalid University, Abha 61913, Saudi Arabia; 3 Department of Family and Community Medicine, College of Medicine, King Khalid University, Abha 61421, Saudi Arabia; 4 Preventive Medicine Program, Aseer Health Cluster, Abha 62523, Saudi Arabia; 5 ERADH and Mental Health Hospital, Jazan Health Cluster, Jazan 82943, Saudi Arabia; 6 Population Health Unit, Jazan Health Affairs, Jazan 82611, Saudi Arabia; 7 Faculty of Medicine, Jazan University, Jazan 45142, Saudi Arabia

Reference 13 has been removed from the original publication [1]. With this correction, the order of some references has been adjusted accordingly. The authors state that the scientific conclusions are unaffected. This correction was approved by the Academic Editor. The original publication has also been updated.
